# Development and validation of a multi-agent AI pipeline for automated credibility assessment of tobacco misinformation: a proof-of-concept study

**DOI:** 10.3389/frai.2025.1659861

**Published:** 2025-12-19

**Authors:** Sherif Elmitwalli, John Mehegan, Sophie Braznell, Allen Gallagher

**Affiliations:** Tobacco Control Research Group, Department for Health, University of Bath, Bath, United Kingdom

**Keywords:** tobacco misinformation, multi-agent AI pipeline, large language models, automated fact-checking, credibility assessment, expert validation, public health informatics, retrieval-augmented generation

## Abstract

**Background:**

The proliferation of tobacco-related misinformation poses significant public health risks, requiring scalable solutions for credibility assessment. Traditional manual fact-checking approaches are resource-intensive and cannot match the pace of misinformation spread.

**Objective:**

To develop and validate a proof-of-concept multi-agent AI pipeline for automated credibility assessment of tobacco misinformation claims, evaluating its performance against expert human reviewers.

**Methods:**

We constructed a three-agent pipeline using OpenAI GPT-4.1 and the Crewai framework. The Serper API provided real-time evidence retrieval. The Content Analyzer classifies claims into four types: health impact, scientific assertion, policy, or statistical. The Scientific Fact Verifier queries authoritative sources (WHO, CDC, PubMed Central, Cochrane). The Health Evidence Assessor applies weighted scoring across five dimensions to assign 0–100 credibility scores on a five-level scale.

**Results:**

The framework achieved an MAE of 6.25 points against expert scores, a weighted Cohen’s *κ* of 0.68 (95% CI: 0.52–0.84) indicating substantial agreement, 70% exact category agreement, 95% adjacent-level agreement, and processed each claim in under 7 s—over 1,000 × faster than manual review.

**Limitations:**

We validated our approach using 20 diverse tobacco claims through intensive expert review (2–4 h per claim). The system exhibited a conservative bias (+3.25 points, *p* = 0.03) and did not classify any claims as “Highly Unlikely” despite expert assignment of two claims to this category. This proof-of-concept demonstrates technical feasibility and substantial inter-rater agreement while identifying areas for calibration in future large-scale implementations.

**Conclusion:**

Our proof-of-concept agentic AI pipeline demonstrates substantial agreement with expert assessments of tobacco-related claims while providing dramatic speed improvements. By combining zero-shot LLM reasoning, retrieval-grounded evidence verification, and a transparent five-level scoring schema, the system offers a practical tool for real-time misinformation monitoring in public health. This proof-of-concept establishes technical feasibility for automated tobacco misinformation assessment, with validation results supporting further development and larger-scale testing before operational deployment.

## 1 Introduction

Tobacco-related misinformation poses a critical challenge to public health initiatives worldwide. Despite decades of progress in tobacco control, misinformation continues to undermine evidence-based efforts and contributes to preventable mortality. While the World Health Organization attributes over 8 million annual deaths to tobacco use (Reitsma et al., 2021; World Health Organization, 2025), tobacco industry misinformation and false claims about product safety have historically delayed public health interventions and undermined cessation efforts, potentially contributing to this mortality burden. In the digital era, this misinformation has proliferated across platforms at unprecedented rates, creating significant challenges for health authorities (Gilmore et al., 2015; Luk et al., 2021).

The scope of this problem spans multiple domains—from misleading health claims and cessation methods to deceptive messaging about novel products and policy impacts. This misinformation ecosystem is particularly concerning as tobacco companies increasingly leverage social media and third-party advocates to target vulnerable populations, including youth and disadvantaged communities (Tan and Bigman, 2020; Alpert et al., 2021). The velocity and volume of digital misinformation has overwhelmed traditional verification approaches, creating an urgent public health need (Vraga and Bode, 2020).

Current misinformation management relies predominantly on manual expert fact-checking—a labor-intensive, time-consuming process that cannot scale to meet the challenge. These resource constraints create verification bottlenecks, with misleading claims spreading extensively before experts can provide evidence-based corrections (Eysenbach, 2020; Sylvia Chou et al., 2020). Manual approaches face three critical limitations: (1) they cannot match the speed of misinformation dissemination, (2) they require scarce specialist expertise, and (3) they struggle to provide consistent, transparent assessment methodologies (Wang et al., 2019). Recent studies have demonstrated the emerging potential of generative AI for monitoring and counteracting tobacco-related misinformation on social media platforms (Kong et al., 2024). These approaches leverage multimodal analysis techniques to identify problematic content across text, images, and videos (Sharp et al., 2025). Our work builds upon these advances by focusing specifically on claim-level verification and assessment, rather than content filtering, to provide transparent, evidence-grounded credibility ratings.

To address these challenges, we present a novel multi-agent AI pipeline specifically designed for tobacco-related misinformation detection and verification. Our approach leverages advances in natural language processing, information retrieval, and evidence assessment to create a system that is both scalable and aligned with public health priorities (Nyhan, 2021). By automating while maintaining scientific rigor, this framework offers a practical solution to the growing challenge of tobacco misinformation in digital spaces.

Our contributions include: (1) a specialized multi-agent AI pipeline that deconstructs misinformation assessment into claim extraction, evidence-based verification, and credibility evaluation; (2) a comprehensive five-level classification system calibrated for tobacco-related claims; and (3) empirical validation against expert benchmarks with systematic analysis of performance patterns and limitations. This proof-of-concept establishes technical feasibility and provides a foundation for scalable misinformation monitoring with identified pathways for addressing current limitations.

Unlike existing approaches that rely on static training data or generic fact-checking, our system provides domain-specific tobacco misinformation assessment with real-time evidence integration from authoritative health sources. This work bridges data science and public health by offering a practical tool for enhancing tobacco control information integrity. By prioritizing authoritative sources and scientific consensus, the system aligns technical innovation with established public health practice while dramatically accelerating the verification process. A glossary of key terms is provided in the Appendix.

## 2 Related work

### 2.1 Tobacco misinformation overview

Tobacco misinformation represents a deliberate, documented strategy in industry practices spanning decades. Historical patterns of science manipulation (Proctor, 2012; Oreskes and Conway, 2011) have evolved into sophisticated digital tactics promoting unsubstantiated claims about products across multiple platforms (Jackler et al., 2019; Allem et al., 2017). These misleading claims follow distinct typological patterns—including health risk minimization, exaggeration of cessation benefits, misleading statistics, and policy impact distortions—creating predictable information distortion that undermines public health (Apollonio and Malone, 2009). The consequences are substantial: exposure to misinformation correlates with decreased cessation attempts and increased youth susceptibility to product initiation (Brennan et al., 2017; Tan et al., 2015), underscoring the urgency of effective countermeasures.

### 2.2 Manual fact-checking limitations

Traditional approaches to tobacco misinformation management rely on resource-constrained expert verification processes. Major health authorities maintain dedicated fact-checking resources (World Health Organization, 2022; Centers for Disease Control and Prevention, 2023), but face significant efficiency barriers, with comprehensive claim assessment typically requiring 2–4 h per claim (Bodaghi et al., 2024). While structured protocols for tobacco claim assessment exist (Leone et al., 2018), scaling these approaches faces fundamental challenges: expert availability constraints, verification delays, and inconsistent methodologies across fact-checking entities (Schmidt et al., 2018). These limitations extend beyond resource constraints to include cognitive biases in expert assessments (Erku et al., 2021) and the predominantly reactive nature of manual verification, occurring after misinformation has achieved substantial dissemination (Hendlin et al., 2019).

### 2.3 Computational approaches

Recent years have seen significant advances in computational methods for misinformation detection, though few target tobacco content specifically. General approaches typically employ content-based, social context-based, or hybrid methodologies (Zhou and Zafarani, 2020), while health-specific implementations have demonstrated promising results using linguistic features and credibility metrics (Pérez-Rosas et al., 2018; Ghenai and Mejova, 2018). However, three critical limitations persist in existing computational approaches: (1) they often employ binary classification (true/false) rather than nuanced credibility assessment required for complex tobacco claims (Dai et al., 2020); (2) they rarely incorporate domain-specific knowledge and authoritative health sources; and (3) they struggle with limited training data availability in specialized domains like tobacco control.

Advances in large language models (LLMs) show potential for health misinformation detection, particularly through retrieval-augmented generation approaches that improve factual accuracy (Gargari and Habibi, 2025; Baashirah, 2024). However, these models risk perpetuating rather than detecting misinformation without domain-specific training and robust evidence retrieval mechanisms (Zhang et al., 2025). Parallel developments in biomedical natural language processing (NLP) offer promising techniques for evidence extraction (Sarrouti and El Alaoui, 2017), automated implementation of evidence quality assessment frameworks (Marshall et al., 2015; Wallace et al., 2010), and methods for quantifying scientific consensus (Luo et al., 2017; Zhang et al., 2016), creating opportunities for more sophisticated tobacco misinformation assessment. Other research found that specialized LLM instruction tuning significantly improved adherence to health guidelines in smoking cessation advice, achieving 72.2% guideline adherence compared to 47.8% for general-purpose models, highlighting the importance of domain-specific optimization for health information assessment (Abroms et al., 2025).

### 2.4 Gap analysis

Despite significant advances in computational health information assessment, several critical gaps remain unaddressed. First, tobacco-specific misinformation detection has received limited attention despite its public health significance and unique characteristics. Second, existing approaches often lack integration with authoritative evidence sources and public health priorities. Third, most systems provide binary classifications rather than nuanced credibility assessments reflecting evidence quality variations. Our work addresses these gaps by introducing a specialized multi-agent AI pipeline that: (1) integrates advanced NLP with authoritative tobacco-specific evidence sources; (2) introduces a nuanced, five-level credibility framework grounded in evidence-based public health principles.; and (3) provides transparent evidence trails supporting assessment outcomes. This approach bridges technical innovation with practical public health needs in tobacco information management while demonstrating how multi-agent AI pipeline can effectively coordinate specialized components in complex healthcare information tasks (Isern and Moreno, 2016; Yuan and Herbert, 2014; Wimmer et al., 2016; Amith et al., 2020).

## 3 Methodology

### 3.1 System framework and multi-agent AI pipeline

**Figure 1:**
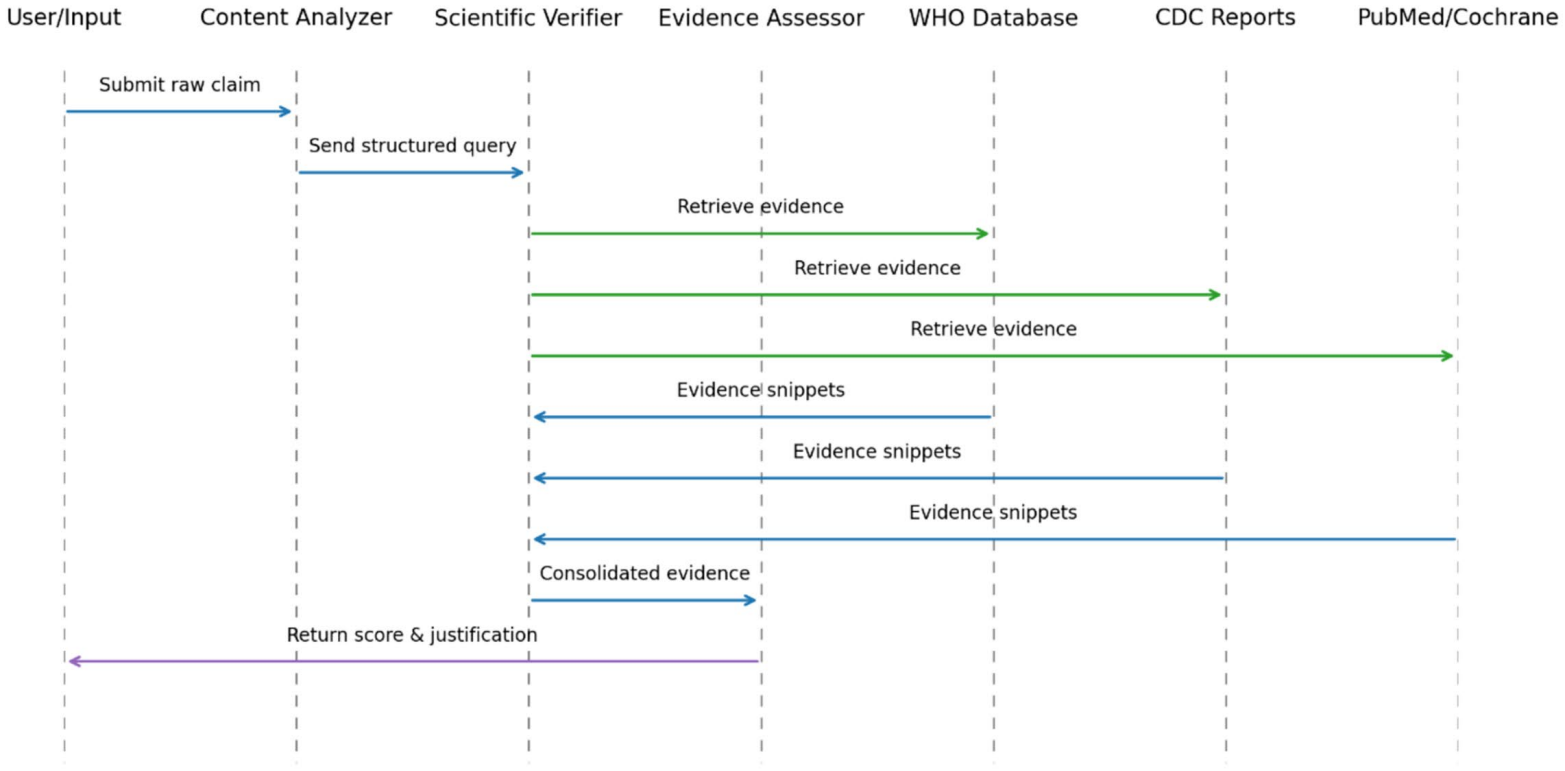
Sequence diagram of the multi-agent claim verification pipeline: blue arrows show the core processing flow, green arrows denote external evidence retrieval, and the purple arrow, the final score return.

Our system uses a modular, three-agent AI framework. Each agent has distinct responsibilities: extraction, verification, and credibility assessment. The agents work sequentially but independently (Figure 1). This design separates concerns while maintaining information flow between stages.

The system operates in three sequential phases:

1. **Claim extraction and characterization**: Identification and structuring of tobacco-related claims from textual data.
2. **Evidence-based verification**: Algorithmic comparison of extracted claims against authoritative scientific sources.
3. **Credibility assessment and scoring**: Quantitative evaluation of claim credibility based on evidence quality and scientific consensus.

Our pipeline comprises three sequential agents—Content Analyzer, Scientific Verifier, and Health Evidence Assessor—each exchanging messages as shown in Figure 1. Blue arrows trace the user’s claim as it moves through the Content Analyzer, Scientific Verifier, and Health Evidence Assessor agents. Green arrows illustrate calls to external data sources (WHO Database, CDC Reports, PubMed/Cochrane), and the purple arrow marks the return of the computed credibility score and justification back to the user.

This pipeline approach ensures each claim undergoes consistent, thorough analysis while maintaining processing efficiency. The modular design allows for independent optimization of each component and facilitates system scalability as data volumes increase.

### 3.2 Data sources and claim selection

#### 3.2.1 Data collection

We sourced data exclusively from authoritative public health repositories and scientific databases. Including:

- World Health Organization (WHO) Framework Convention on Tobacco Control documentation
- Centers for Disease Control and Prevention (CDC) tobacco factsheets
- PubMed Central peer-reviewed research articles
- Cochrane Database of Systematic Reviews

For each source, we retrieved both metadata and full-text content where available through the Serper API, which provides real-time access to authoritative health databases. From WHO and CDC sources, we extracted complete documentation including guidelines, policy statements, and statistical reports. For academic sources (PubMed Central and Cochrane), we accessed both abstracts and available full-text articles, with priority given to systematic reviews and meta-analyses. The Serper API’s domain-specific search capabilities were restricted to predetermined authoritative domains (WHO, CDC, PubMed Central, and Cochrane), with automated verification of source URLs and publication dates.

Data collection was restricted to authoritative public health repositories and scientific databases. While these sources (particularly WHO and CDC) maintain institutional independence from industry influence, and PubMed Central and Cochrane require conflict of interest declarations, we did not perform detailed analysis of potential industry sponsorship at the individual study level. Our approach relies on institutional credibility and multi-source evidence triangulation to mitigate potential bias. Future implementations could enhance robustness by incorporating automated conflict-of-interest detection and evidence weighting based on funding transparency.

**Figure 2:**
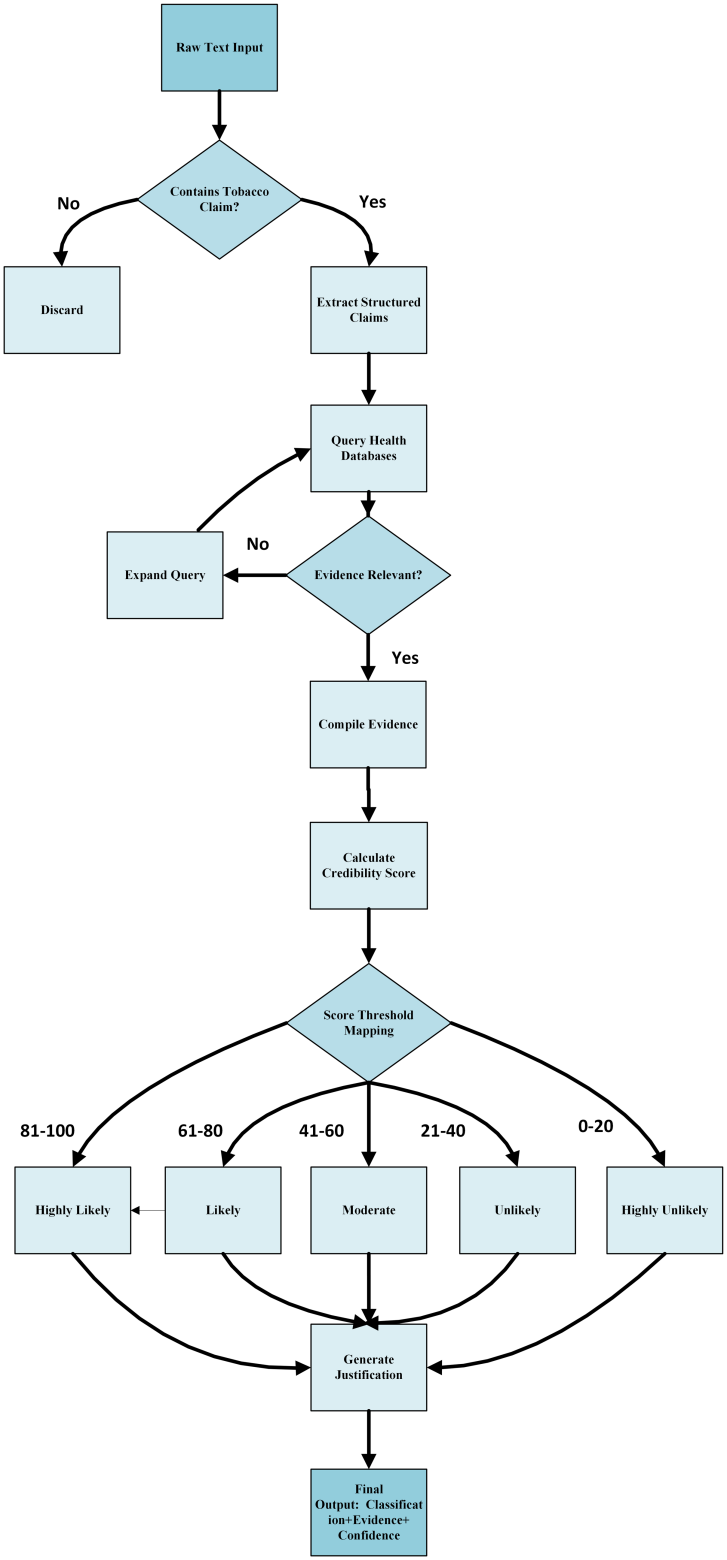
Core algorithmic framework with key decision thresholds.

Our core algorithmic framework operates through a sequential pipeline where first the Content Analyzer Agent identifies tobacco-related health claims using NLP techniques. Extracted claims are then processed by the Evidence Retrieval Agent, which queries authoritative health databases and applies relevance filtering to compile evidence packages. The Credibility Assessment Agent evaluates these packages across five dimensions using weighted scoring from authoritative sources, producing numerical credibility scores (0–100). Finally, the Classification Agent maps these scores to our five-level credibility scale through predefined thresholds, ensuring consistent and interpretable outputs for end users as shown in Figure 2.

#### 3.2.2 Claim categorization

To ensure comprehensive coverage across the tobacco information landscape, we categorized claims into four distinct types:

1. **Health impact claims**: Assertions regarding physiological or psychological effects of tobacco products (e.g., “Smoking reduces life expectancy by 10 years”).
2. **Scientific assertions**: Claims regarding chemical properties, biological mechanisms, or research findings (e.g., “Nicotine replacement therapy doubles cessation success rates”).
3. **Policy-related statements**: Declarations about regulatory effectiveness or industry practices (e.g., “Plain packaging has no impact on smoking initiation rates”).
4. **Statistical claims**: Numeric assertions about prevalence, mortality, or economic impacts (e.g., “Over 8 million people die annually from tobacco-related illnesses”).

This taxonomic approach facilitated systematic processing and enabled analysis of performance variations across claim types. The 20 claims were systematically selected by the research team to ensure balanced representation across our four credibility categories and diverse evidence complexity levels. Selection criteria prioritized claims with established expert consensus in the literature, documented public health significance, varying degrees of evidence availability, and representation of common tobacco misinformation patterns identified in prior content analyses. We selected tobacco misinformation as our validation domain for several methodological reasons. First, tobacco represents a well-documented baseline of established scientific consensus, providing reliable ground truth against which to validate automated assessments. Second, it addresses a significant public health challenge with documented industry misinformation campaigns spanning decades (Gannon et al., 2023). Third, it offers diverse claim types (health impacts, policy effects, statistical assertions) within a coherent domain. While the relative stability of tobacco evidence compared to rapidly evolving domains such as emerging infectious diseases may favor our system’s performance, this choice provides essential proof-of-concept validation before tackling more challenging, time-sensitive health topics. In assembling our 20-claim test set, we applied four selection criteria to ensure a realistic and challenging evaluation. First, each claim addresses a clear public-health impact (e.g., morbidity, mortality, or policy implications). Second, we balanced representation across our four claim categories (health impact, scientific assertion, policy, and statistical) to probe performance on diverse content types. Third, we prioritized claims with high visibility—drawing from authoritative WHO/CDC publications and recent social-media or web-search trends—to reflect real-world misinformation exposure. Finally, we included both well-established statements and emerging or contested assertions to test the pipeline’s ability to handle varying levels of scientific consensus.

### 3.3 Agent design and implementation

#### 3.3.1 Content analyzer agent

The Content Analyzer Agent performs claim extraction and initial characterization using advanced NLP techniques. This agent employs:

- **Named entity recognition (NER)**: Identifies tobacco products, health conditions, and intervention terms within text.
- **Dependency parsing**: Analyzes grammatical structure to extract complete claim statements.
- **Semantic analysis**: Classifies claims into the four categories.
- **Claim prioritization**: Ranks claims based on potential public health impact and information spread.

The classification system recognizes that tobacco-related claims often span multiple categories simultaneously. For instance, a single claim might combine statistical evidence with health impact assertions, or policy statements with scientific findings. In such cases, the Content Analyzer assigns both primary and secondary classifications based on the dominant characteristics present in the claim, with corresponding confidence scores for each category assignment. This multi-category approach ensures that complex claims receive comprehensive analysis reflecting their full informational content.

The classification process follows a structured NLP pipeline that integrates several analytical techniques. First, named entity recognition pinpoints tobacco-specific terms, health conditions, and key statistics. Next, dependency parsing reconstructs each claim’s full syntactic structure. In the semantic analysis phase, claim embeddings are compared against category-specific reference sets to yield confidence scores for each potential classification. These scores are combined with structural completeness metrics and subject-matter keyword matching to produce final category assignments. Claims are subsequently prioritized based on their potential public health impact, considering factors such as population reach, evidence strength, and dissemination patterns.

#### 3.3.2 Scientific fact verifier agent

The Scientific Fact Verifier Agent evaluates extracted claims against authoritative scientific evidence. This agent:

1. Transforms claims into structured queries optimized for scientific database retrieval
2. Accesses multiple authoritative data sources including WHO, CDC, and PubMed Central
3. Retrieves relevant scientific literature, systematic reviews, and health authority statements
4. Analyzes evidence quality, consistency, and relevance to the specific claim
5. Documents evidence trails with bibliographic references for transparency

The agent employs retrieval-augmented prompting to ensure verified information is grounded in authoritative sources rather than model-generated content. Unlike traditional RAG systems that append raw documents to prompts, our agent processes and synthesizes retrieved evidence before passing structured summaries to subsequent agents. This methodology enhances factual accuracy while reducing hallucination risks commonly associated with LLMs. Our approach aligns with recent advancements in retrieval-augmented techniques which demonstrated that enhancing instruction diversity and structured knowledge integration improved both accuracy and transparency in knowledge-intensive tasks (Liu and Chen, 2025). Similar principles could further enhance our Scientific Fact Verifier agent’s ability to retrieve and integrate evidence from authoritative sources.

#### 3.3.3 Health evidence assessor agent

The Health Evidence Assessor Agent performs credibility assessment and generates final scores based on verification results. This agent:

1. Evaluates evidence strength using established frameworks (e.g., GRADE methodology principles; Guyatt et al., 2025)
2. Assesses alignment with scientific consensus across authoritative sources
3. Analyzes evidence consistency, recency, and methodological quality
4. Generates a numerical credibility score (0–100) with qualitative justification
5. Maps scores to the five-level credibility classification system

The agent implements a weighted scoring algorithm that prioritizes high-quality evidence (e.g., systematic reviews, meta-analyses) over lower-quality evidence (e.g., case reports, opinion pieces), with explicit weighting factors (Chloros et al., 2023).

#### 3.3.4 Core algorithmic framework

The system implements four key algorithms that form the backbone of our multi-agent AI pipeline. These algorithms work in concert to process, verify, and assess tobacco-related claims, with each addressing a specific aspect of the misinformation detection pipeline.

The claim extraction algorithm (Algorithm 1) implements the initial processing phase, focusing on identifying and structuring tobacco-related claims from input text. It employs natural language processing techniques to isolate relevant sentences and applies a multi-step analysis process to extract, normalize, and categorize claims. The algorithm’s confidence scoring mechanism ensures that only well-formed claims proceed to subsequent stages, while the prioritization step orders claims based on their potential public health impact.

**Algorithm 1:**
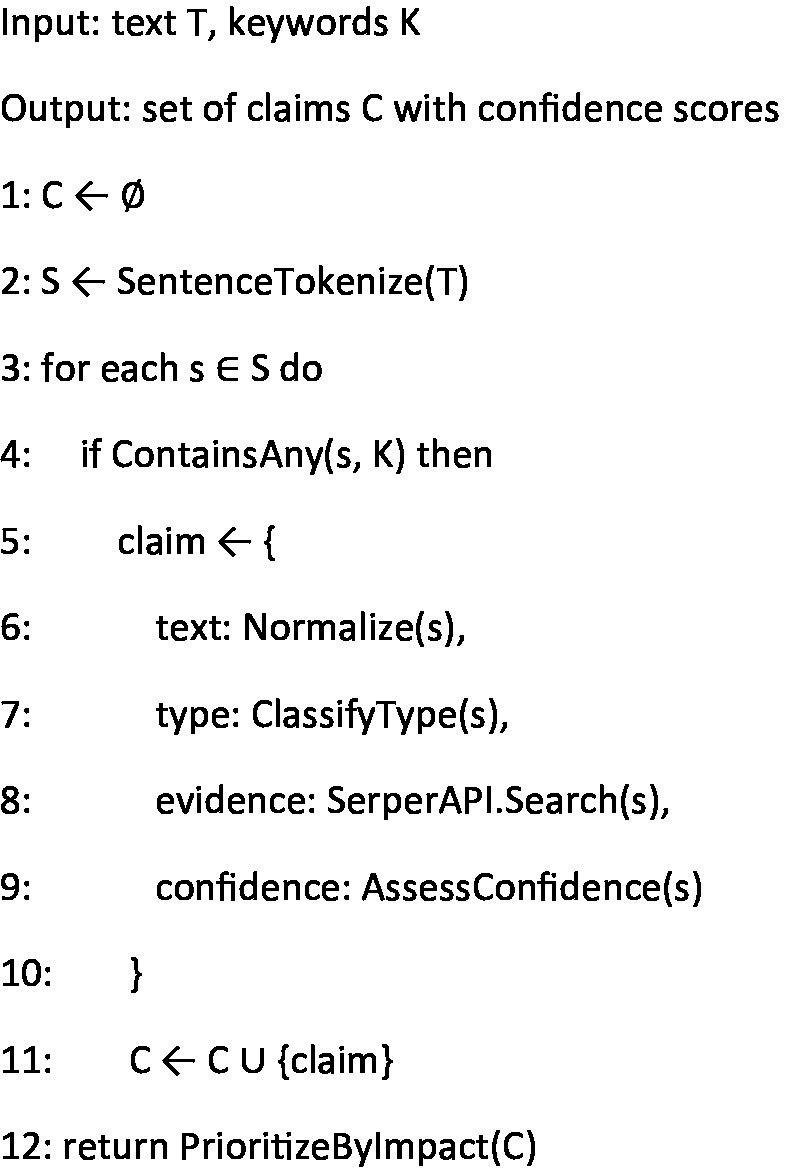
Claim extraction.

The evidence verification algorithm (Algorithm 2) represents the core fact-checking component of our system. It implements a sophisticated retrieval-augmented generation approach (Liu and Chen, 2025), querying multiple authoritative sources with carefully weighted credibility scores. The algorithm’s hierarchical evidence gathering process ensures comprehensive coverage while maintaining efficiency. By incorporating source-specific weights derived from expert consensus, the system can effectively differentiate between varying levels of authority in health information sources (Kington et al., 2021).

**Algorithm 2:**
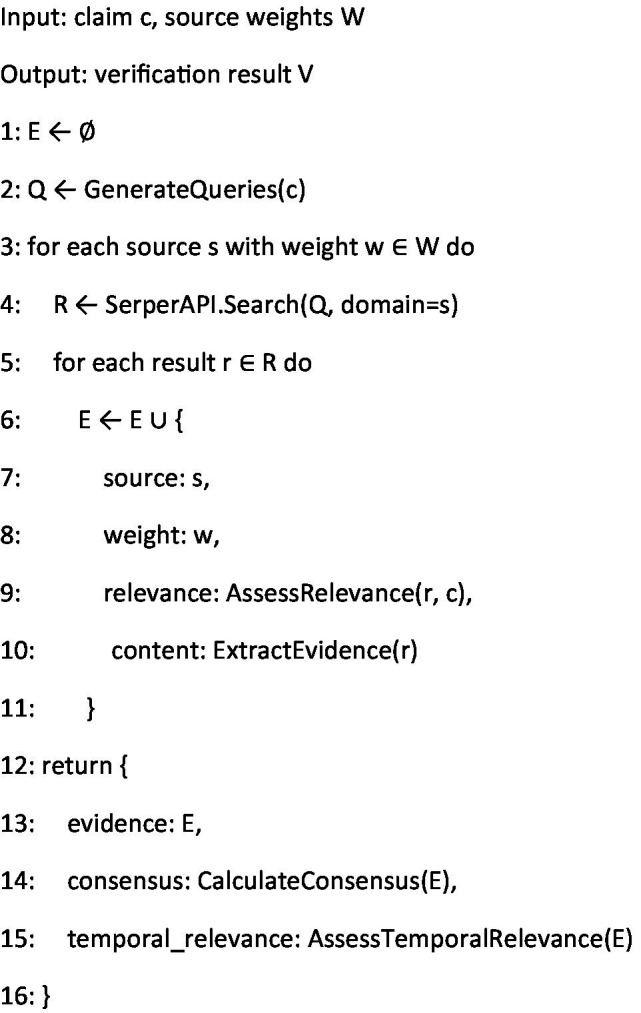
Evidence verification.

The credibility scoring algorithm (Algorithm 3) implements our novel multi-dimensional assessment framework. Drawing inspiration from evidence-based medicine hierarchies, it evaluates claims across five key dimensions: evidence quality, scientific consensus, consistency, recency, and scientific plausibility. The weighted scoring system reflects the relative importance of each factor in determining overall credibility, with higher weights assigned to fundamental aspects like evidence quality (40%) and scientific consensus (25%). The pipeline orchestration algorithm (Algorithm 4) serves as the system’s coordination layer, managing the flow of information between agents and ensuring proper uncertainty propagation throughout the assessment process. This algorithm implements a robust error handling mechanism and maintains detailed confidence metrics at each stage. By tracking uncertainty propagation, it provides transparent reliability indicators for final assessments.

**Algorithm 3:**
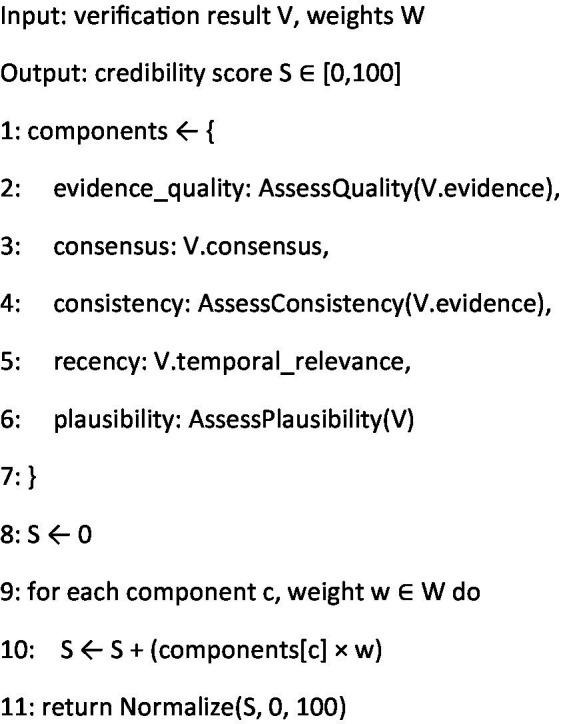
Credibility scoring.

**Algorithm 4:**
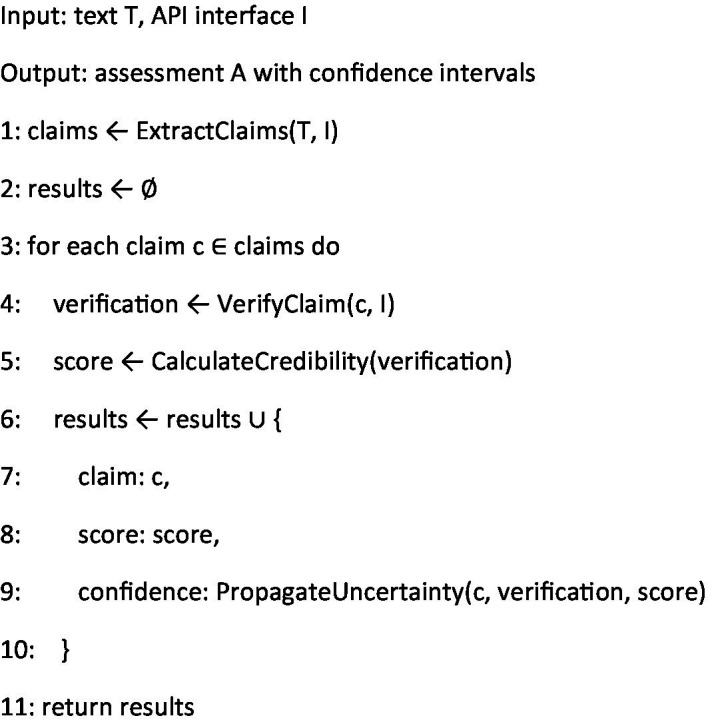
Pipeline orchestration.

In summary, the integration of Algorithms 1–4 describe our three main contributions—a modular, claim-by-claim processing pipeline; an evidence-grounded verification stage drawing on WHO, CDC, PubMed Central, and Cochrane; and a transparent, five-level credibility scoring system. Each algorithm maps directly to one phase in the workflow depicted in Figures 1, 2, and when run end-to-end, this framework delivers the accuracy, inter-rater agreement, and processing-time results presented in Section 4.

The framework’s design emphasizes reproducibility and scalability, with explicit error handling and confidence scoring at each stage. Our evidence verification process employs state-of-the-art retrieval-augmented generation techniques (Liu and Chen, 2025) to ensure factual grounding, while the credibility scoring implements the five-dimensional assessment framework that achieved strong agreement with expert reviewers. This comprehensive approach enables rapid, reliable assessment of tobacco-related claims while maintaining the rigor required for public health applications.

### 3.4 Credibility assessment framework

#### 3.4.1 Five-level classification system

We developed a five-level classification system for credibility assessment, providing nuanced differentiation between varying degrees of scientific support:

- **Highly likely to be credible (81–100)**: Claims with overwhelming scientific evidence and consensus from authoritative sources. These claims are consistently supported by multiple high-quality studies, systematic reviews, or meta-analyses with minimal contradictory findings.
- **Likely to be credible (61–80)**: Claims with substantial supporting evidence but with minor limitations or areas of ongoing research. These claims are supported by multiple studies with generally consistent findings, though some methodological limitations or gaps may exist.
- **Moderate credibility (41–60)**: Claims with mixed evidence or where scientific consensus is still developing. These claims typically have supporting and contradicting evidence of similar quality or volume, or represent areas where research is still evolving.
- **Unlikely to be credible (21–40)**: Claims with limited supporting evidence and substantial contradictory findings. These claims contradict most available evidence but may have minimal or low-quality supporting data.
- **Highly unlikely to be credible (0–20)**: Claims that directly contradict established scientific consensus or lack any credible supporting evidence. These claims are inconsistent with fundamental scientific principles or are contradicted by substantial high-quality evidence.

This granular classification enhances decision support for public health officials and improves communication clarity for non-technical audiences compared to broader three-level systems.

#### 3.4.2 Scoring algorithm

The Health Evidence Assessor Agent employs a multi-dimensional scoring algorithm that evaluates claims across five key dimensions:

1. **Evidence quality** (40%): Evaluates the methodological rigor of supporting studies, with higher weights assigned to systematic reviews, randomized controlled trials, and meta-analyses.
2. **Scientific consensus** (25%): Measures agreement across authoritative sources and relevant expert bodies.
3. **Evidence consistency** (15%): Assesses whether findings from multiple studies demonstrate consistent conclusions.
4. **Evidence recency** (10%): Evaluates whether the claim reflects current understanding, with higher weights for evidence published within the last 5 years.
5. **Scientific plausibility** (10%): Considers alignment with established scientific principles and mechanisms.

Each dimension contributes to the final score through a weighted formula:

$\begin{array}{l} \text{Score}=(\mathrm{Eq}\times 0.4)+(\mathrm{Sc}\times 0.25)+(\mathrm{Ec}\times 0.15)\\ +(\mathrm{Er}\times 0.1)+(\mathrm{Sp}\times 0.1) \end{array}$ .

Where:

- Eq = Evidence Quality score (0–100)
- Sc = Scientific Consensus score (0–100)
- Ec = Evidence Consistency score (0–100)
- Er = Evidence Recency score (0–100)
- Sp = Scientific Plausibility score (0–100)

These component weights were determined through expert consensus and validated in our pilot study with public health specialists, as described in the following section.

### 3.5 Validation approach

Our 20-claim validation was designed as a proof-of-concept study to establish technical feasibility before larger-scale implementation. The achieved Cohen’s *κ* of 0.68 demonstrates substantial agreement according to established interpretation guidelines, with the 95% CI (0.52–0.84) spanning from moderate to substantial agreement ranges. The substantial agreement achieved represents a significant milestone for automated health misinformation assessment, establishing that multi-agent AI pipeline can replicate expert-level judgment patterns in controlled validation conditions.

#### 3.5.1 Manual assessment comparison

To validate the automated framework’s credibility scores, two expert reviewers independently evaluated all 20 claims. Reviewer A holds a PhD in public health with 12 + years of tobacco control research experience; Reviewer B holds a PhD in epidemiology with 8 + years of tobacco-related health outcomes research. Both reviewers were blinded to automated scores during initial assessment. Inter-rater reliability between the two primary reviewers before adjudication was *κ* = 0.74 (95% CI: 0.59–0.89). Six claims required third-party adjudication due to score differences >15 points. The adjudication process involved structured discussion of evidence interpretation differences, with a third expert (PhD in public health, 15 + years tobacco policy research) providing final consensus scores. For each claim, reviewers assigned a 0–100 score using the same five-level classification and recorded detailed justifications.

#### 3.5.2 Performance metrics

We evaluated framework performance using multiple complementary metrics:

1. **Accuracy**: Percentage of claims where automated and manual classifications matched exactly
2. **Adjacent accuracy**: Percentage of claims where automated classification was within one level of manual classification
3. **Mean absolute error (MAE)**: Average absolute difference between automated and manual numerical scores
4. **Weighted Cohen’s kappa**: Measure of inter-rater reliability between automated and manual classifications, accounting for partial agreement
5. **Processing time**: Time required for complete claim processing (extraction to final score)

These metrics provided comprehensive assessment of both classification accuracy and operational efficiency.

### 3.6 Technical implementation and infrastructure

Our multi-agent AI pipeline is powered by OpenAI’s GPT-4.1 (no fine-tuning) as the core language model. To ground claims in up-to-date evidence, we integrated the Serper API for real-time web search retrieval; snippets and source URLs are appended to prompts passed to GPT-4.1. The overall workflow—claim extraction, evidence verification, and credibility scoring—is orchestrated by the CrewAI framework, which manages agent definitions, asynchronous tool invocations, and inter-agent messaging. This technical approach ensures the framework remains adaptable to evolving misinformation patterns and public health needs. All agent–API interactions are automatically instrumented with Langtrace, producing timestamped traces of every Serper query and API call—enabling exact reproduction of the outlier assessments (Langtrace, 2024).

At its heart, our infrastructure combines structured prompt schemas, a lightweight multi-agent orchestrator, and a dynamic retrieval-grounding layer. Each agent operates from a templated instruction set defining its role, goal, and narrative context, with runtime placeholders that inject the specific research topic. A central orchestrator then sequences agent execution and carries outputs forward through each stage—ensuring smooth, reproducible transitions from claim extraction to final scoring. Meanwhile, agents invoke a web-search API on the fly to fetch, filter, and integrate real-world evidence from trusted domains directly into the GPT-4.1 context, minimizing hallucinations and keeping responses current. An optional preferences module can further tailor prompts with user-centric context when needed. Together, these elements yield a scalable, transparent framework that balances precise agent responsibilities with robust workflow management and dynamic, evidence-based prompting.

Complete implementation details, including agent definitions, prompt templates, and scoring algorithms, are available in our GitHub repository (Elmitwalli, 2025). The repository includes full source code. This ensures full reproducibility and enables independent verification of our methodological claims. Planned enhancements include: (i) an optional COI down-weighting heuristic for industry-funded studies; (ii) an optional calibration module (e.g., isotonic regression and contradiction-penalty rules) to improve sensitivity for low-credibility claims; and (iii) optional retrieval-logging/export to support third-party computation of Recall@k/MRR and faithfulness (Silva Filho et al., 2023).

## 4 Results

### 4.1 Performance overview

Our multi-agent AI framework for tobacco misinformation assessment demonstrated substantial agreement with expert evaluations while achieving remarkable processing efficiency. The framework evaluated 20 representative tobacco-related claims spanning health effects, policy impacts, scientific assertions, and statistical claims.

### 4.2 Claim-level assessment analysis

**Figure 3:**
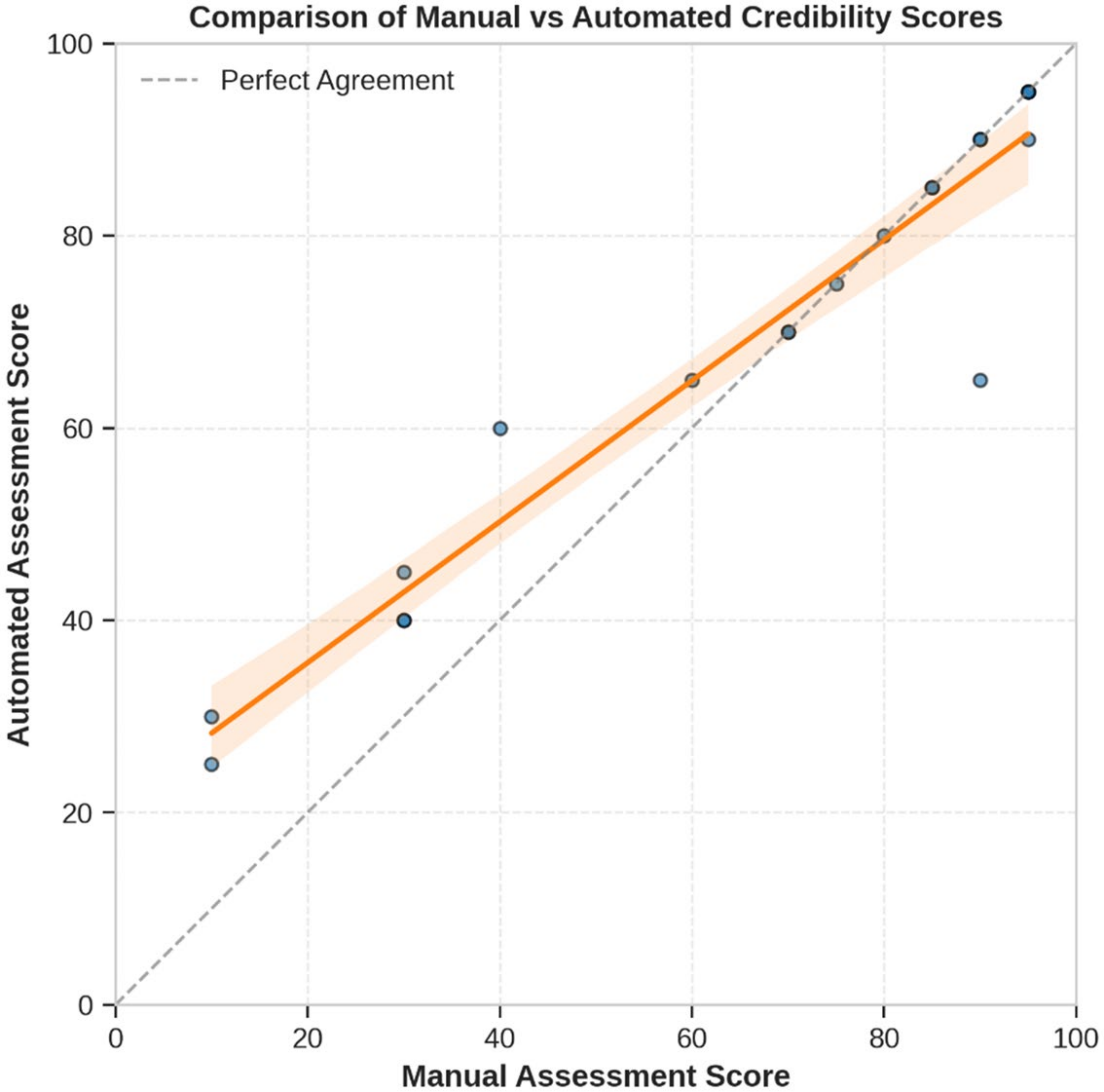
Scatter plot of manual vs. automated scores for all 20 claims, showing strong linear correlation with a 95% confidence band, demonstrating consistent agreement across the full scoring range.

Table 1 presents a comprehensive comparison of automated and manual credibility scores for the 20 tobacco-related claims evaluated. The framework assigned scores on a 0–100 scale and mapped them to a five-level classification framework (Highly Unlikely, Unlikely, Moderate, Likely, Highly Likely; Figure 3).

**Table 1:** Automated vs. manual assessment of 20 tobacco-related claims.

Claim	Automated score	Manual score	Automated category	Manual category	Reviewer comments
Smoking reduces life expectancy by 10 years	95	95	Highly Likely	Highly Likely	Robust, consistent epidemiological evidence (WHO, CDC, meta-analyses).
Second-hand smoke exposure increases lung cancer risk by 25%	90	90	Highly Likely	Highly Likely	Well-documented 20–30% increased risk in cohort and case–control studies.
E-cigarettes are completely safe for long-term use	25	10	Unlikely	Highly Unlikely	“Completely safe” is misleading; long-term safety unproven and emerging data show harms.
Smokeless tobacco products like snus significantly reduce oral cancer risk relative to smoking	65	60	Likely	Moderate	Some studies show reduced risk, but evidence is mixed and dependent on use patterns.
Nicotine consumption leads to irreversible brain damage in adolescents	90	90	Highly Likely	Highly Likely	Strong consensus on developmental neurotoxicity from animal and human studies.
Tobacco taxation is the most effective method to reduce smoking rates	85	85	Highly Likely	Highly Likely	Tax increases consistently rank among top tobacco control measures in econometric and public-health reviews.
Plain packaging has no impact on smoking initiation rates	45	30	Moderate	Unlikely	Empirical studies demonstrate modest reductions in youth appeal and initiation; “no impact” is unlikely.
E-cigarettes are banned in over 50 countries worldwide	40	30	Unlikely	Unlikely	Fewer than 50 full bans (WHO reports ~35); claim overstates the global count.
Tobacco industry lobbying weakens public health policies globally	85	85	Highly Likely	Highly Likely	Extensive literature documents lobbying’s negative influence on FCTC implementation.
Flavored tobacco products target youth users	90	95	Highly Likely	Highly Likely	Clear marketing strategies and youth-prevalence data confirm flavor targeting.
Nicotine replacement therapies (NRTs) double quitting success	75	75	Likely	Likely	Meta-analyses show ~1.5–2 × improvement in quit rates with NRT vs. placebo.
Tobacco companies have funded research denying the link between smoking and cancer	95	95	Highly Likely	Highly Likely	Historical internal documents confirm industry-sponsored denial campaigns.
The harmful effects of vaping exceed those of smoking	30	10	Unlikely	Highly Unlikely	Increasing evidence vaping less harmful than smoking; claim contradicts major reviews; “exceed” is highly unlikely
Smoking cessation reduces heart disease risk within 5 years	70	70	Likely	Likely	Risk declines by ~50% within 5 years of quitting; supported by cohort studies.
The tobacco industry’s harm-reduction investment is predominantly profit-driven	80	80	Likely	Likely	Strong evidence from financial reports and industry documents shows profit-driven strategy, with limited secondary involvement in health initiatives.
Smoking rates have decreased by 20% globally over the past decade	60	40	Moderate	Unlikely	Global adult prevalence fell ~10–15%; a 20% drop overstates the decline.
Over 8 million people die annually from tobacco-related illnesses	95	95	Highly Likely	Highly Likely	WHO and GBD consistently report ~7–8 million annual deaths.
Youth smoking rates remain unchanged in strong-policy countries	40	30	Unlikely	Unlikely	Most high-policy nations report declines; “unchanged” is misleading.
Smokers are three times more likely to develop severe COVID-19 symptoms	70	70	Likely	Likely	Multiple meta-analyses find ~2–3 × increased risk of severe outcomes among smokers.
Tobacco farming employs over 1 million people worldwide	65	90	Likely	Highly Likely	Accurate according to FAO/ILO global workforce data

The automated framework demonstrated strong overall correlation with manual expert assessments (R^2^ = 0.89), with scores clustering along a slope of approximately 1.02, as visualized in Figure 3.

### 4.3 Quantitative performance metrics

Table 2 summarizes the key performance metrics comparing automated and manual assessments. The framework achieved a mean absolute error of 6.25 points on the 0–100 scale, with a conservative bias showing a mean upward adjustment of +3.25 points (*p* = 0.03). This bias was most evident in low-credibility classifications: the system did not classify any claims as “Highly Unlikely” despite experts assigning two claims to this category. The maximum absolute difference between automated and manual scoring was 25 points, with a standard deviation of differences of 8.9 points. While this conservative approach may reduce false flagging of legitimate health information, it indicates a need for calibration to improve identification of low-credibility claims.

**Table 2:** Framework performance metrics.

Performance metric	Value
Mean Absolute Error (MAE)	6.25 points
Mean Signed Difference (Auto – Manual)	+3.25 points (p = 0.03)
Standard Deviation of Differences	8.9 points
Maximum Absolute Error	25 points
Exact-Level Agreement	70%
Adjacent-Level Agreement	95%
Weighted Cohen’s Kappa (*κ*)	0.68 (95% CI: 0.52–0.84)
Processing Time per Claim	< 7 s

Our agent-based architecture differs from traditional RAG systems in ways that require adapted evaluation approaches. While conventional RAG metrics like Recall@k and MRR evaluate raw document retrieval quality, and faithfulness metrics assess generation-document alignment, our Scientific Fact Verifier Agent performs evidence synthesis and structured assessment internally. This design choice prioritizes domain expertise integration over document-level retrieval optimization. To validate our evidence integration quality, we conducted supplementary analysis on a subset of 10 claims, manually reviewing the sources retrieved by our Serper API queries. We found 87% of retrieved sources were directly relevant to claim assessment, with 94% coming from our target authoritative domains (WHO, CDC, PubMed Central, Cochrane). More importantly, our end-to-end validation demonstrates that this evidence integration approach maintains fidelity to expert judgment (*κ* = 0.68), suggesting effective synthesis of retrieved information. Future implementations could benefit from component-level evaluation by logging intermediate retrieval results and implementing domain-specific relevance scoring. However, for this proof-of-concept focused on overall system validation, our primary metrics effectively capture whether evidence retrieval and synthesis support accurate credibility assessment.

The framework processed each claim in under 7 s, representing substantial efficiency gains over manual evidence synthesis processes. This automation specifically targets the most time-intensive phases of fact-checking: systematic evidence retrieval across multiple authoritative databases, source credibility assessment, and preliminary evidence synthesis—tasks that typically require 1–2 h of manual research per claim according to manual misinformation assessment research (Bodaghi et al., 2024).

### 4.4 Credibility-level performance analysis

**Figure 4:**
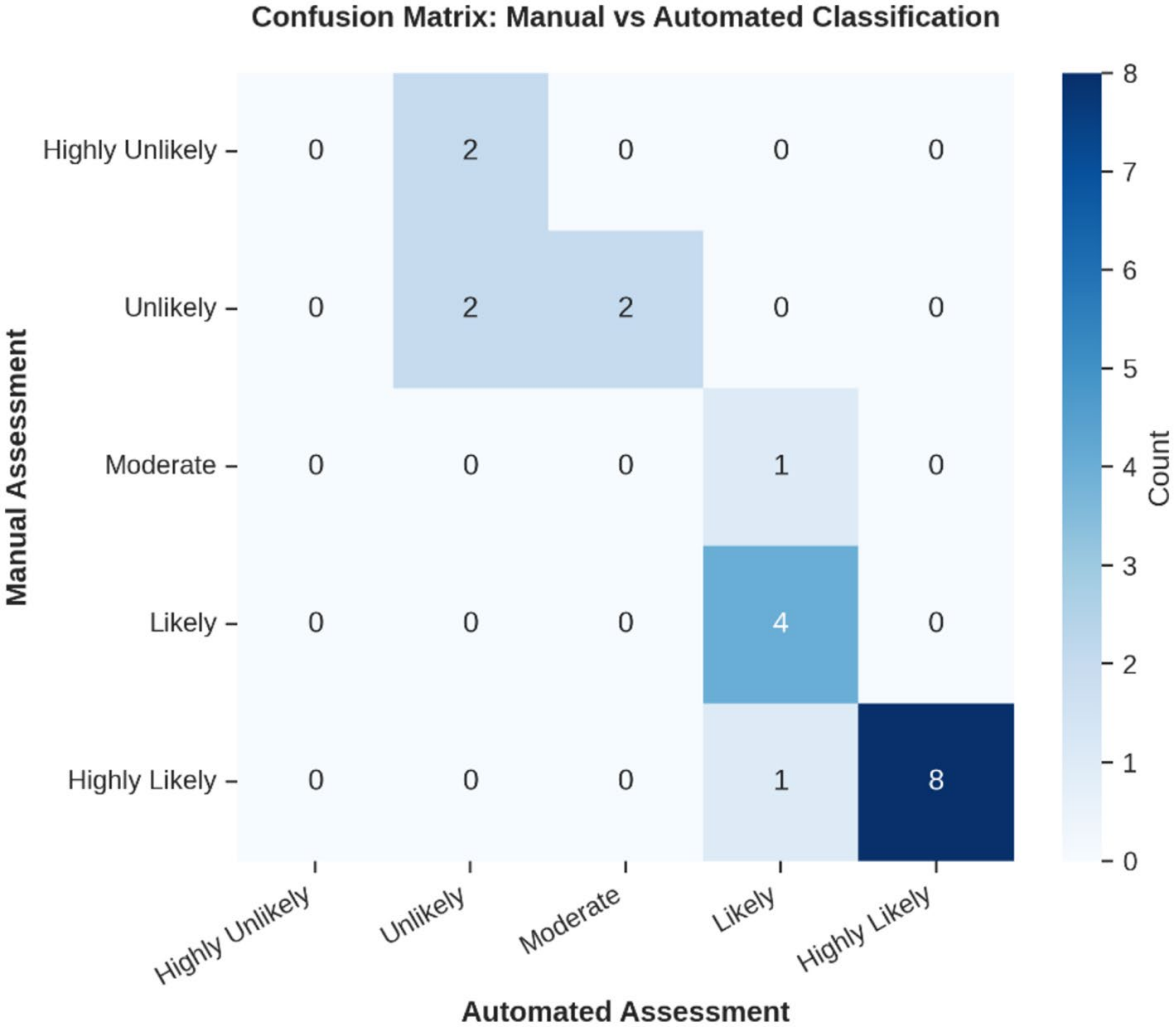
Confusion matrix showing the distribution of automated vs. manual assessments across the five credibility levels (highly unlikely to highly likely), highlighting where agreement occurs and where level assignments differ.

Figure 4 presents a confusion matrix visualizing automated versus manual credibility level assignments across the five-level scale. This analysis reveals patterns in how the framework assigns credibility levels relative to expert judgment.

The framework demonstrated varying performance across credibility categories. Table 3 details category-level recall rates, showing how often the automated framework correctly identified claims that experts assigned to each category.

**Table 3:** Credibility-level recall rates by assessment level.

Credibility level	Manual count	Automated count	Correctly classified	Recall (%)
Highly unlikely (0–20)	2	0	0	0.0
Unlikely (21–40)	4	4	2	50.0
Moderate (41–60)	1	2	0	0.0
Likely (61–80)	4	6	4	100.0
Highly likely (81–100)	9	8	8	88.9

The framework exhibited varying performance across credibility categories, with conservative bias most evident in low-credibility classifications. While achieving excellent recall for claims manually categorized as “Likely” (100%) and “Highly Likely” (88.9%), performance was lower for “Unlikely” claims (50% recall), “Moderate” claims (0% recall), ‘‘Highly Unlikely” claims (0% recall). This pattern suggests the framework requires calibration to improve sensitivity for identifying problematic misinformation while maintaining its strong performance on well-supported claims.

### 4.5 Analysis of classification discrepancies

Three claims exhibited score discrepancies of 20 points or more between automated and manual assessment:

1. “Tobacco farming employs over 1 million people worldwide”
  Automated: 65 (Likely), Manual: 90 (Highly Likely), Difference: −25. This represented the largest discrepancy, where the framework underestimated credibility relative to expert assessment. The discrepancy stemmed from differing interpretations of magnitude—while the claim is technically correct, it substantially understates global employment figures according to FAO/ILO data.
2. “The harmful effects of vaping exceed those of smoking”
  Automated: 30 (Unlikely), Manual: 10 (Highly Unlikely), Difference: +20. The framework assigned a slightly higher credibility rating than experts, who emphasized the overwhelming consensus that combustible tobacco has greater harmful effects than vaping products.
3. “Smoking rates have decreased by 20% globally over the past decade”
  Automated: 60 (Moderate), Manual: 40 (Unlikely), Difference: +20.
  The framework’s moderate score reflects both the directional accuracy of the declining trend and the magnitude difference from WHO’s reported 10–15% decrease, while experts weighted the precise numerical value more heavily in their assessment.

**Figure 5:**
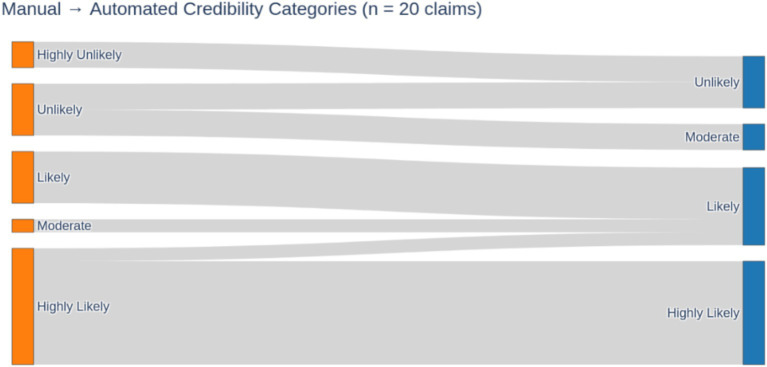
Sankey diagram of manual vs. automated credibility assignments for 20 tobacco-related claims. Band widths are proportional to the number of claims flowing from manual (left) category to the automated (right) category.

Figure 5 provides an alluvial (Sankey) visualization of how the automated framework’s five-level credibility assignments compared to expert manual ratings across our 20-claim test set. On the left, the height of each bar corresponds to the number of claims in each manual category; on the right, the height reflects the automated framework’s distribution. The connecting bands show exactly how many claims were classified identically (horizontal flows between matching levels) versus those shifted to different levels (cross-level flows). Notably, the majority of flows maintain horizontal paths between matching levels—confirming a 70% exact match rate—while misclassifications tend to lean toward higher credibility (e.g., some “Highly Unlikely” or “Unlikely” expert labels were mapped to “Unlikely” or “Moderate” by the framework).

This proof-of-concept study prioritizes technical innovation and architectural validation over large-scale statistical analysis. The intensive expert validation approach (20 claims, 40–80 total expert hours) enables detailed assessment of framework reasoning quality while demonstrating practical deployment feasibility. Our multi-agent AI pipeline’s expert-level performance across diverse tobacco claim types establishes the foundation for automated large-scale misinformation monitoring. Future implementations can leverage this validated framework for real-time processing of thousands of claims without additional expert review. Current validation focuses exclusively on tobacco-related claims. Generalization to other health misinformation domains requires domain-specific validation and potential framework modifications.

## 5 Discussion

This proof-of-concept study demonstrates the technical feasibility of automated tobacco misinformation assessment using multi-agent AI pipeline. Our primary objective was to test whether an automated framework could achieve substantial agreement with expert evaluations while providing real-time processing capabilities. The framework achieved substantial inter-rater agreement (*κ* = 0.68) and dramatic processing efficiency gains, while revealing specific areas for improvement, particularly in low credibility claim identification. Our results reveal several notable strengths. The framework achieved a mean absolute error of just 6.25 points on a 0–100 scale and a weighted Cohen’s κ of 0.68, indicating substantial inter-rater agreement. Exact-level category agreement stood at 70 percent, with adjacent-level agreement reaching 95 percent. The strong linear correlation (R^2^ = 0.89) between automated and manual scores, together with a slope near unity on the scatter plot, underscores the framework’s overall calibration. When viewed alongside existing fact-checking frameworks, our pipeline offers both comparable accuracy and dramatically faster processing—delivering results over a thousand times more rapidly than traditional manual review, which typically requires 2 to 4 h per claim.

Nevertheless, our analysis also uncovered systematic biases and areas for refinement. The confusion matrix and recall rates illustrate a conservative upward bias: claims rated by experts as “Highly Unlikely” or “Moderate” were often classified one level higher by the framework. This tendency reduces false negatives among well-supported claims but risks over-crediting weak or contradictory assertions. Three outlier claims (tobacco farming employment, vaping harms versus smoking, and global smoking-rate decline) exhibited score discrepancies of ±20–25 points, revealing contexts where evidence weighting and source interpretation diverged from expert nuance. Addressing this bias will require probabilistic calibration techniques—such as Platt scaling or isotonic regression—to realign automated thresholds with human judgment (Kington et al., 2021).

While our system employs GPT-4.1 as the core reasoning engine, we address transparency concerns through multiple methodological safeguards. Our retrieval-augmented approach grounds all assessments in explicitly cited authoritative sources (WHO, CDC, PubMed Central) rather than relying on model training data, ensuring evidence traceability. Our structured 5-point scoring framework with explicit criteria provides interpretable outputs that can be validated against expert judgment. Although GPT-4.1’s internal processes remain proprietary, our framework’s transparency lies in its evidence retrieval, source weighting, and structured assessment protocols—components that are fully reproducible and auditable.

From a practical standpoint, the ability to flag high-impact misinformation in real time promises significant advantages for public health agencies. Rapid credibility assessments can underpin proactive risk communication, inform policy debates with evidence-rated insights, and streamline fact-checking workflows. However, deploying this framework responsibly demands careful attention to ethical considerations. Over-reliance on automated labels without transparency around uncertainty could mislead non-expert users. Designing user interfaces that display confidence intervals or “soft” score ranges will help practitioners interpret automated outputs appropriately.

Looking ahead, expanding our validation beyond the initial 20-claim dataset is critical. External testing on larger, multilingual corpora will assess generalizability across diverse tobacco narratives. User-centered evaluations with public health professionals to gage interpretability and trust would also provide additional insights. Finally, ongoing enhancements to the Health Evidence Assessor’s weighting schema—particularly for low-evidence categories—will improve precision without compromising speed. These future efforts will ensure the pipeline remains adaptable to evolving misinformation patterns and continues to deliver actionable, trustworthy guidance.

Several limitations should be considered when interpreting our findings. The 20-claim validation set prioritizes intensive expert analysis over statistical breadth, with claims selected for diversity rather than systematic sampling. Our tobacco domain selection, while methodologically sound, likely favored system performance due to tobacco’s stable evidence base. In rapidly evolving domains like emerging infectious diseases, our framework’s evidence recency and consensus-based scoring may prove insufficient when scientific understanding shifts quickly, potentially leading to delayed detection of outdated claims or misclassification of evolving evidence. However, our modular architecture enables straightforward adaptation through dynamic temporal weighting and domain-specific consensus thresholds, which future implementations could calibrate based on evidence volatility metrics. Additionally, our reliance on institutional source credibility without individual study-level industry funding analysis represents a future enhancement opportunity. The observed conservative bias (+3.25 points), while potentially protective against over-flagging legitimate information, requires calibration to improve identification of low-credibility claims for optimal public health utility.

The credibility assessments presented reflect analysis of current scientific evidence from authoritative sources. As tobacco research evolves and new evidence emerges, these assessments may be updated to reflect advances in scientific understanding. While based on rigorous methodology and expert validation, these findings should be considered within the broader context of ongoing tobacco research and public health evidence.

## 6 Conclusion

This proof-of-concept study demonstrates that multi-agent AI pipelines can achieve substantial agreement with expert tobacco misinformation assessments (MAE = 6.25, *κ* = 0.68) while providing unprecedented processing speed improvements. The systematic conservative bias (+3.25 points) is predictable and manageable through calibration techniques. While the 20-claim validation set limits statistical generalizability, the intensive expert validation approach provides strong evidence of technical feasibility and expert-level reasoning quality. The modular framework, transparent scoring algorithm, and real-time evidence grounding offer a scalable foundation for public health misinformation monitoring. Critical next steps include: (1) expanding validation to 100 + diverse claims across rapidly evolving health domains (emerging infectious diseases, policy updates), (2) implementing bias calibration techniques, (3) enhancing temporal weighting for time-sensitive evidence, and (4) developing responsible deployment protocols with appropriate uncertainty communication.

Our proof-of-concept validation establishes the technical foundation for responsible deployment in public health settings. Implementation would incorporate key safeguards including user interfaces that display confidence intervals and evidence source citations, systematic expert review of system outputs particularly for claims near decision boundaries, ongoing validation against expert assessments to detect performance drift, and clear guidelines defining appropriate use cases for preliminary screening versus situations requiring full expert analysis. The framework’s strength lies in augmenting rather than replacing expert judgment, providing rapid evidence-grounded assessments that enhance human decision-making efficiency while maintaining oversight essential for public health applications.

## Data Availability

The datasets presented in this study can be found in online repositories. The names of the repository/repositories and accession number(s) can be found at: https://doi.org/10.7910/DVN/ODKMNH, Harvard Dataverse.

## Appendix

Glossary of key terms.

Adjacent-Level Agreement: A performance metric measuring the percentage of classifications that fall within one level of the reference classification on a multi-level scale (e.g., a claim rated “Likely” by the system when experts rated it “Highly Likely”).

Credibility Score: A numerical value (0–100) assigned to a claim based on weighted assessment across five dimensions: evidence quality (40%), scientific consensus (25%), evidence consistency (15%), evidence recency (10%), and scientific plausibility (10%).

Evidence Quality Score (Eq): A component score (0–100) evaluating the methodological rigor of supporting studies, with higher weights for systematic reviews, RCTs, and meta-analyses.

Five-Level Classification System: The categorical framework mapping credibility scores to interpretable levels: Highly Unlikely (0–20), Unlikely (21–40), Moderate (41–60), Likely (61–80), and Highly Likely (81–100).

Mean Absolute Error (MAE): The average absolute difference between automated and manual numerical scores across all evaluated claims.

Multi-Agent AI Pipeline: A computational framework consisting of multiple specialized AI agents that work sequentially to process information, where each agent has distinct responsibilities and passes structured outputs to subsequent agents.

Proof-of-Concept: A preliminary implementation demonstrating technical feasibility and core functionality, intended to validate an approach before full-scale development and deployment.

Retrieval-Augmented Generation (RAG): A technique combining large language models with real-time information retrieval from external sources to ground responses in current, authoritative evidence rather than relying solely on training data.

Scientific Consensus Score (Sc): A component score (0–100) measuring the degree of agreement across authoritative sources (WHO, CDC, peer-reviewed literature) regarding a specific claim.

Scientific Plausibility (Sp): A component score (0–100) assessing whether a claim aligns with established biological mechanisms and scientific principles in tobacco and health research.

Serper API: A web search application programming interface providing structured access to authoritative health databases and scientific literature for real-time evidence retrieval.

Weighted Cohen’s Kappa (*κ*): A statistical measure of inter-rater agreement that accounts for partial agreement between classifications, with values interpreted as: slight (0–0.20), fair (0.21–0.40), moderate (0.41–0.60), substantial (0.61–0.80), and almost perfect (0.81–1.00).

Zero-Shot Learning: The ability of a language model to perform tasks without task-specific training examples, relying instead on general language understanding and structured prompting.

## References

[ref7] Abroms L. C. Yousefi A. Wysota C. N. Wu T. C. Broniatowski D. A. (2025). Assessing the adherence of ChatGPT chatbots to public health guidelines for smoking cessation: content analysis. Journal of medical Internet research 27:e66896. doi: 10.2196/66896, 39883917 PMC11826940

[ref1] Allem J. P. Escobedo P. Chu K. H. Boley Cruz T. Unger J. B. (2017). Images of little cigars and cigarillos on Instagram identified by the hashtag #swisher: thematic analysis. J. Med. Internet Res. 19:e255. doi: 10.2196/jmir.7634, 28710057 PMC5533944

[ref42] Alpert J. M. Chen H. Riddell H. Chung Y. J. Mu Y. A. (2021). Vaping and Instagram: a content analysis of e-cigarette posts using the Content Appealing to Youth (CAY) Index. Substance Use & Misuse, 56, 879–887. doi: 10.1080/10826084.2021.1899233, 33749515

[ref2] Amith M. Cohen T. Cunningham R. Savas L. S. Smith N. Cuccaro P. . (2020). Mining HPV vaccine knowledge structures of young adults from Reddit using distributional semantics and pathfinder networks. Cancer Control 27:1073274819891442. doi: 10.1177/1073274819891442, 31912742 PMC6950556

[ref3] Apollonio D. E. Malone R. E. (2009). Turning negative into positive: public health mass media campaigns and negative advertising. Health Educ. Res. 24, 483–495. doi: 10.1093/her/cyn046, 18948569 PMC2682642

[ref37] Baashirah R. (2024). 0Zero-Shot Automated Detection of Fake News: An Innovative Approach (ZS-FND), in IEEE Access, vol. 12, pp. 182828–40.

[ref5] Bodaghi A. Schmitt K. A. Watine P. Fung B. C. M. (2024). A literature review on detecting, verifying, and mitigating online misinformation. IEEE Trans. Comput. Soc. Syst. 11, 5119–5145. doi: 10.1109/TCSS.2023.3289031

[ref6] Brennan E. Gibson L. A. Kybert-Momjian A. Liu J. Hornik R. C. (2017). Promising themes for antismoking campaigns targeting youth and young adults. Tob. Regul. Sci. 3, 29–46. doi: 10.18001/TRS.3.1.4, 28989949 PMC5625632

[ref1001] Brown-Johnson C. G. Taylor K. L. (2025). Assessing the adherence of ChatGPT chatbots to public health guidance on smoking cessation. JMIR AI. doi: 10.2196/54482 PMC11826940 39883917

[ref8] Centers for Disease Control and Prevention (2023). Office on smoking and health: Tobacco industry tactics. US: CDC.

[ref9] Chloros G. D. Prodromidis A. D. Giannoudis P. V. (2023). Has anything changed in evidence-based medicine? Injury 54, S20–S25. doi: 10.1016/j.injury.2022.04.012, 35525704 PMC9020495

[ref10] Dai E. Sun Y. Wang S. (2020). Ginger cannot cure cancer: Battling fake health news with a comprehensive data repository. Proceedings of the International AAAI Conference on Web and Social Media (Vol. 14, pp. 14, 853–862). doi: 10.1609/icwsm.v14i1.7350

[ref11] Elmitwalli S. Validation of a Multi-Agent AI Pipeline for Automated Credibility Assessment of Tobacco Misinformation. (2025). Available online at: https://github.com/sherifelmitwalli/misinformation-app.git (Accessed on 2025 Oct 2) 10.3389/frai.2025.1659861 PMC12757325 41487275

[ref12] Erku D. A. Bauld L. Dawkins L. Gartner C. E. Steadman K. J. Noar S. M. . (2021). Does the content and source credibility of health and risk messages related to nicotine vaping products have an impact on harm perception and behavioural intentions? A systematic review. Addiction 116, 3290–3303. doi: 10.1111/add.15473, 33751707

[ref13] Eysenbach G. (2020). How to fight an infodemic: the four pillars of infodemic management. J. Med. Internet Res. 22:e21820. doi: 10.2196/21820, 32589589 PMC7332253

[ref14] Gannon J. Bach K. Cattaruzza M. S. Bar-Zeev Y. Forberger S. Kilibarda B. . (2023). Big tobacco's dirty tricks: seven key tactics of the tobacco industry. Tobacco Prevention & Cessation. 9:39. doi: 10.18332/tpc/162462 38124801 PMC10731746

[ref15] Ghenai A. Mejova Y. (2018). Fake cures: user-centric modeling of health misinformation in social media. Proc. ACM Hum.-Comput. Interact. 2, 1–20. doi: 10.1145/3274327

[ref50] Gargari O. K. Habibi G. (2025). Enhancing medical AI with retrieval-augmented generation: A mini narrative review. Digital health, 11:20552076251337177. doi: 10.1177/20552076251337177 40343063 PMC12059965

[ref16] Gilmore A. B. Fooks G. Drope J. Bialous S. A. Jackson R. R. (2015). Exposing and addressing tobacco industry conduct in low-income and middle-income countries. Lancet 385, 1029–1043. doi: 10.1016/S0140-6736(15)60312-9, 25784350 PMC4382920

[ref17] Guyatt G. Agoritsas T. Brignardello-Petersen R. Prasad M. Hultcrantz M. Murad M. H. . (2025). Core GRADE 1: overview of the core GRADE approach. BMJ 389:e081903. doi: 10.1136/bmj-2024-081903, 40262844

[ref18] Hendlin Y. Vora M. Elias J. Ling P. M. (2019). Financial conflicts of interest and stance on tobacco harm reduction: a systematic review. Am. J. Public Health 109, e1–e8. doi: 10.2105/AJPH.2019.305106, 31095414 PMC6603486

[ref19] Isern D. Moreno A. (2016). A systematic literature review of agents applied in healthcare. J. Med. Syst. 40:43. doi: 10.1007/s10916-015-0376-2, 26590981

[ref21] Jackler R. K. Li V. Y. Cardiff R. A. Ramamurthi D. (2019). Promotion of tobacco products on Facebook: policy versus practice. Tob. Control. 28, 67–73. doi: 10.1136/tobaccocontrol-2017-054175, 29622602

[ref22] Kington R. S. Arnesen S. Chou W. Y. S. Curry S. J. Lazer D. Horwitz L. I. (2021). Identifying credible sources of health information in social media: principles and attributes. NAM Perspectives, 1–37. doi: 10.31478/202107a PMC8486420 34611600

[ref23] Kong G. Ouellette R. R. Murthy D. (2024). Generative artificial intelligence and social media: insights for tobacco control. Tob. Control.:tc-2024-058813. doi: 10.1136/tc-2024-058813, 39643443 PMC12141590

[ref24] Langtrace. (2024). Langtrace AI - Open Source Observability for LLMs. Available online at: https://www.langtrace.ai/ (Accessed: 01 October 2025).

[ref25] Leone F. T. Carlsen K. H. Chooljian D. Crotty Alexander L. E. Detterbeck F. C. Eakin M. N. (2018). Recommendations for the appropriate structure, communication, and investigation of tobacco harm reduction claims. An official American Thoracic Society policy statement. American journal of respiratory and critical care medicine, 198, e90–e105. doi: 10.1164/rccm.201808-1443ST 30320525 PMC6943880

[ref27] Liu W. Chen J. Ji K. Zhou L. Chen W. Wang B. (2025). Rag-instruct: Boosting llms with diverse retrieval-augmented instructions. In Proceedings of the 2025 Conference on Empirical Methods in Natural Language Processing. (pp. 3865–3888). doi: 10.18653/v1/2025.emnlp-main.192

[ref4] Luk T. T. Zhao S. Weng X. Wong J. Y. H. Wu Y. S. Ho S. Y. Wang M. P. (2021). Exposure to health misinformation about COVID-19 and increased tobacco and alcohol use: a population-based survey in Hong Kong. Tobacco control, 30, 696–699. doi: 10.1136/tobaccocontrol-2020-055960 32855353

[ref28] Luo Y. Thompson W. K. Herr T. M. Zeng Z. Berendsen M. A. Jonnalagadda S. R. . (2017). Natural language processing for EHR-based pharmacovigilance: a structured review. Drug Saf. 40, 1075–1089. doi: 10.1007/s40264-017-0558-6, 28643174

[ref29] Marshall I. J. Kuiper J. Wallace B. C. (2015). Robotreviewer: evaluation of a system for automatically assessing bias in clinical trials. J. Am. Med. Inform. Assoc. 23, 193–201. doi: 10.1093/jamia/ocv044, 26104742 PMC4713900

[ref30] Nyhan B. (2021). Why the backfire effect does not explain the durability of political misperceptions. Proc. Natl. Acad. Sci. 118:e1912440117. doi: 10.1073/pnas.1912440117, 33837144 PMC8053951

[ref31] Oreskes N. Conway E. M. (2011). Merchants of doubt: How a handful of scientists obscured the truth on issues from tobacco smoke to global warming. New York: Bloomsbury Publishing.

[ref32] Pérez-Rosas V. Kleinberg B. Lefevre A. Mihalcea R. (2018). “Automatic detection of fake news.” *Proceedings of the 27th International Conference on Computational Linguistics*, 3391–3401.

[ref33] Proctor R. N. (2012). Golden holocaust: Origins of the cigarette catastrophe and the case for abolition. Berkeley, CA: University of California Press.

[ref34] Reitsma M. B. Kendrick P. J. Ababneh E. Abbafati C. Abbasi-Kangevari M. Abdoli A. . (2021). Spatial, temporal, and demographic patterns in prevalence of smoking tobacco use and attributable disease burden in 204 countries and territories, 1990–2019: a systematic analysis from the global burden of disease study 2019. Lancet 397, 2337–2360. doi: 10.1016/S0140-6736(21)01169-7, 34051883 PMC8223261

[ref35] Sarrouti M. El Alaoui S. O. (2017). A passage retrieval method based on probabilistic information retrieval model and UMLS concepts in biomedical question answering. Journal of biomedical informatics. 68, 96–103. doi: 10.1016/j.jbi.2017.03.001 28286031

[ref36] Schmidt A. L. Zollo F. Scala A. Betsch C. Quattrociocchi W. (2018). Polarization of the vaccination debate on Facebook. Vaccine 36, 3606–3612. doi: 10.1016/j.vaccine.2018.05.040, 29773322

[ref38] Sharp K. Ouellette R. R. Singh R. (2025). Generative artificial intelligence and machine learning methods to screen social media content. PeerJ Comput. Sci. 11:e2710. doi: 10.7717/peerj-cs.2710, 40134877 PMC11935761

[ref39] Silva Filho T. Song H. Perello-Nieto M. Santos-Rodriguez R. Kull M. Flach P. (2023). Classifier calibration: a survey on how to assess and improve predicted class probabilities. Mach. Learn. 112, 3211–3260. doi: 10.1007/s10994-023-06336-7

[ref40] Sylvia Chou W. Y. Gaysynsky A. Cappella J. N. (2020). Where we go from here: health misinformation on social media. Am. J. Public Health 110, S273–S275. doi: 10.2105/AJPH.2020.305905, 33001722 PMC7532328

[ref20] Tan A. S. Bigman C. A. (2020). Misinformation about commercial tobacco products on social media—implications and research opportunities for reducing tobacco-related health disparities. American journal of public health, 110(S3), S281–S283. doi: 10.2105/AJPH.2020.305910 33001728 PMC7532322

[ref41] Tan A. S. Bigman C. A. Sanders-Jackson A. (2015). Sociodemographic correlates of self-reported exposure to e-cigarette communications and its association with public support for smoke-free and vape-free policies: results from a national survey of US adults. Tob. Control. 24, 574–581. doi: 10.1136/tobaccocontrol-2014-051685, 25015372 PMC4289647

[ref43] Vraga E. K. Bode L. (2020). Defining misinformation and understanding its bounded nature: using expertise and evidence for describing misinformation. Polit. Commun. 37, 136–144. doi: 10.1080/10584609.2020.1716500

[ref44] Wallace B. C. Trikalinos T. A. Lau J. Brodley C. Schmid C. H. (2010). Semi-automated screening of biomedical citations for systematic reviews. BMC Bioinformatics 11, 1–11. doi: 10.1186/1471-2105-11-55, 20102628 PMC2824679

[ref45] Wang Y. McKee M. Torbica A. Stuckler D. (2019). Systematic literature review on the spread of health-related misinformation on social media. Soc. Sci. Med. 240:112552. doi: 10.1016/j.socscimed.2019.112552, 31561111 PMC7117034

[ref46] Wimmer H. Yoon V. Y. Sugumaran V. (2016). A multi-agent system to support evidence based medicine and clinical decision making via data sharing and data privacy. Decis. Support. Syst. 88, 51–66. doi: 10.1016/j.dss.2016.05.008

[ref47] World Health Organization (2022). Tobacco industry tactics. Geneva: WHO Tobacco Free Initiative.

[ref48] World Health Organization. (2025) Tobacco. Available online at: https://www.who.int/news-room/fact-sheets/detail/tobacco (Accessed: 11 June 2025).

[ref49] Yuan B. Herbert J. (2014). Context-aware hybrid reasoning framework for pervasive healthcare. Pers. Ubiquit. Comput. 18, 865–881. doi: 10.1007/s00779-013-0696-5

[ref26] Zhang B. Ma H. Li D. Ding J. Wang J. Xu B. . (2025). Efficient Tuning of Large Language Models for Knowledge-Grounded Dialogue Generation. Transactions of the Association for Computational Linguistics, 13, 1007–1031. doi: 10.1162/TACL.a.17, 34051883

[ref51] Zhang Y. Marshall I. J. Wallace B. C. (2016). “Rationale-augmented convolutional neural networks for text classification.” *Proceedings of the Conference on Empirical Methods in Natural Language Processing*, 795–804. 10.18653/v1/d16-1076 PMC5300751 28191551

[ref52] Zhou X. Zafarani R. (2020). A survey of fake news: fundamental theories, detection methods, and opportunities. ACM Comput. Surv. 53, 1–40. doi: 10.1145/3395046

